# Crystal structure and Hirshfeld surface analysis of 4,4′-(propane-1,3-diyl)bis(4*H*-1,2,4-triazol-1-ium) penta­fluorido­oxidovanadate(V)

**DOI:** 10.1107/S205698902000585X

**Published:** 2020-05-01

**Authors:** Ganna A. Senchyk, Andrey B. Lysenko, Harald Krautscheid, Kostiantyn V. Domasevitch

**Affiliations:** a Inorganic Chemistry Department, National Taras Shevchenko University of Kyiv, Volodymyrska Str. 64/13, 01601 Kyiv, Ukraine; b Institute of Inorganic Chemistry, Leipzig University, Johannisallee 29, D-04103 Leipzig, Germany

**Keywords:** crystal structure, penta­fluorido­oxidovanadate(V), 1,2,4-triazole, hydrogen bonding, Hirshfeld surface

## Abstract

In the structure of the title salt, (C_7_H_12_N_6_)[VOF_5_], second-order Jahn–Teller distortion of the coordination octa­hedra around V ions is reflected by coexistence of short V—O bonds [1.5767 (12) Å] and *trans*-positioned long V—F bonds [2.0981 (9) Å], with four equatorial V—F distances being inter­mediate in magnitude [1.7977 (9)–1.8913 (9) Å]. Hydrogen bonding of the anions is restricted to F-atom acceptors only, with particularly strong N–H⋯F inter­actions [N⋯F = 2.5072 (15) Å] established by axial and *cis*-positioned equatorial F atoms. Hirshfeld surface analysis indicates that the most important inter­actions are overwhelmingly H⋯F/F⋯H, accounting for 74.4 and 36.8% of the contacts for the individual anions and cations, respectively. Weak CH⋯F and CH⋯N bonds are essential for generation of three-dimensional structure.

## Abstract 2

In the structure of the title salt, second-order Jahn–Teller distortion of the coordination octa­hedra around V ions is reflected by coexistence of short V—O bonds and *trans*-positioned long V—F bonds, with four equatorial V—F distances being inter­mediate in magnitude. Hydrogen bonding of the anions is restricted to F-atom acceptors only, with particularly strong N–H⋯F inter­actions [N⋯F = 2.5072 (15) Å] established by axial and *cis*-positioned equatorial F atoms.

## Chemical context   

Significant second-order Jahn–Teller distortions are inherent to the coordination octa­hedra of the [*M* ^v^OF_5_]^2−^ series (*M* ^v^ = V, Nb, Ta) of ions (Ok *et al.*, 2006 ▸; Welk *et al.*, 2002 ▸). The resulting polar symmetry of the anions could be exploited as the origin of bulk polarity when imprinted on the structures of non-centrosymmetric coordination and hydrogen bonded solids (Halasyamani, 2010 ▸). Such supra­molecular synthesis with oxofluoride building blocks extends existing approaches for the development of non*-*centrosymmetric crystals, which attract significant inter­est for electro-optical applications (Gautier & Poeppelmeier, 2013 ▸).

One can anti­cipate that [VOF_5_]^2−^ systems will show this effect to a particular extent since the vanadium ions experience a much larger out-of-centre displacement towards an apical O-ligand compared with their Nb and Ta analogues (Ok *et al.*, 2006 ▸). This feature generates a larger dipole moment as well as mitigating against orientational disorder of the anions in crystal structures (Sharko *et al.*, 2018 ▸). However, the supra­molecular behaviour of the [VOF_5_]^2−^ anions is less predictable and it is strikingly different from that of the most extensively examined Nb and Ta systems. Welk *et al.* (2000 ▸) noted the very weak O-coordinating ability of the [VOF_5_]^2−^ anions serving as F-donor ligands only but the hydrogen-bond acceptor ability of the O atoms is less addressed. Distal inter­actions of the C—H⋯O type are relevant to the structure of (H_2_bipy)[VOF_5_] (bipy is 4,4′-bi­pyridine; Gautier *et al.*, 2015 ▸), but surprisingly, no hydrogen bonding at all was observed for the O atoms in (H_2_En)[VOF_5_] (En is ethyl­enedi­amine; Rieskamp & Mattes, 1976 ▸). In addition, the possible competitiveness of the O atoms with respect to other weak hydrogen-bond acceptors does not appear to have been considered so far.
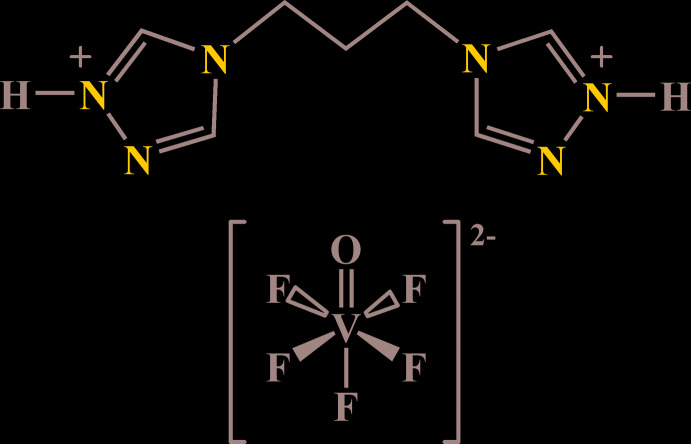


With this in mind, we now describe the synthesis and structure of the title salt, (C_7_H_12_N_6_^2+^)·[VOF_5_]^2−^, which gives insight into the hydrogen-bonding behaviour of [VOF_5_]^2−^ anions when combined with the bitopic nitro­gen-rich 4,4′-(propane-1,3-diyl)bis(4*H*-1,2,4-triazol-1-ium) dication. This cation provides different kinds of hydrogen-bond donor sites complemented by triazole-N acceptors, which are relevant to many types of coordination and hydrogen-bonded systems (Senchyk *et al.*, 2017 ▸; Lysenko *et al.*, 2010 ▸).

## Structural commentary   

**Figure 1:**
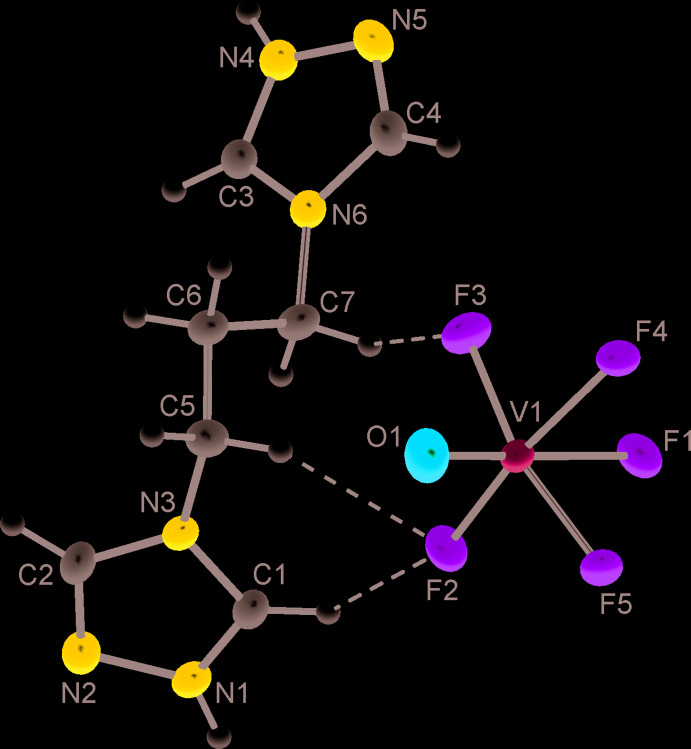
The mol­ecular structure of the title compound with displacement ellipsoids drawn at the 50% probability level. Dotted lines indicate weak C—H⋯F hydrogen bonding.

The mol­ecular structure of the title compounds is shown in Fig. 1 ▸. The distorted coordination octa­hedra around the V ions comprise very short V1—O1 bonds of 1.5767 (12) Å and long bonds with *trans*-positioned F1 ligands [V1—F1 = 2.0981 (9) Å], which define the local polar axis of the anion. Four equatorial V—F bonds [mean 1.8295 (9) Å, Table 1 ▸] are inter­mediate in length. That the anion geometry is sensitive to the hydrogen-bond environment is evidenced by the elongation of the V1—F4 bonds [1.8913 (9) Å], with the F4 atoms involved in a strong N—H⋯F inter­action (Table 2 ▸). The central ion deviates from the centroid of its six ligand atoms by *d* = 0.242 Å towards the O-vertex. This is reminiscent of the geometrical features of the [VOF_5_]^2−^ anions in the salts with (H_2_bipy)^2+^ (*d* = 0.268 Å; Gautier *et al.*, 2015 ▸) and (H_2_En)^2+^ cations (*d* = 0.272 Å; Rieskamp & Mattes, 1976 ▸).

The main geometrical parameters of the organic cations are very similar to those of the parent 1,3-propyl­enebitriazole ligand in complexes with metal ions (Senchyk *et al.*, 2017 ▸). The dicationic structure, as the result of protonation of the N1 and N4 sites, is best reflected by differentiation of the angles involving the N atoms in the two triazolium rings: C—N(H)—N = 111.17 (12) and 111.79 (11)° *versus* C—N—N(H) = 103.46 (12) and 104.11 (12)° (Table 1 ▸). A similar effect is known for the isoelectronic neutral pyrazole ring (Gospodinov *et al.*, 2020 ▸). The protonation also results in a certain shortening of the N—N bonds [1.362 (2) Å], as may be compared with N—N = 1.3918 (15) Å for the neutral and non-coordinated triazole rings in the adamantane derivative (Lysenko *et al.*, 2019 ▸). The methyl­ene linkage adopts a *trans–gauche* conformation with the corresponding torsion angles C5—C6—C7—N6 of −171.58 (12)° and N3—C5—C6—C7 of −63.73 (17)°. A diversity of metal complexes suggest nearlys equal occurrence of *trans–gauche* and all-*trans* sequences for the present moiety (Senchyk *et al.*, 2017 ▸).

## Supra­molecular features   

**Figure 2:**
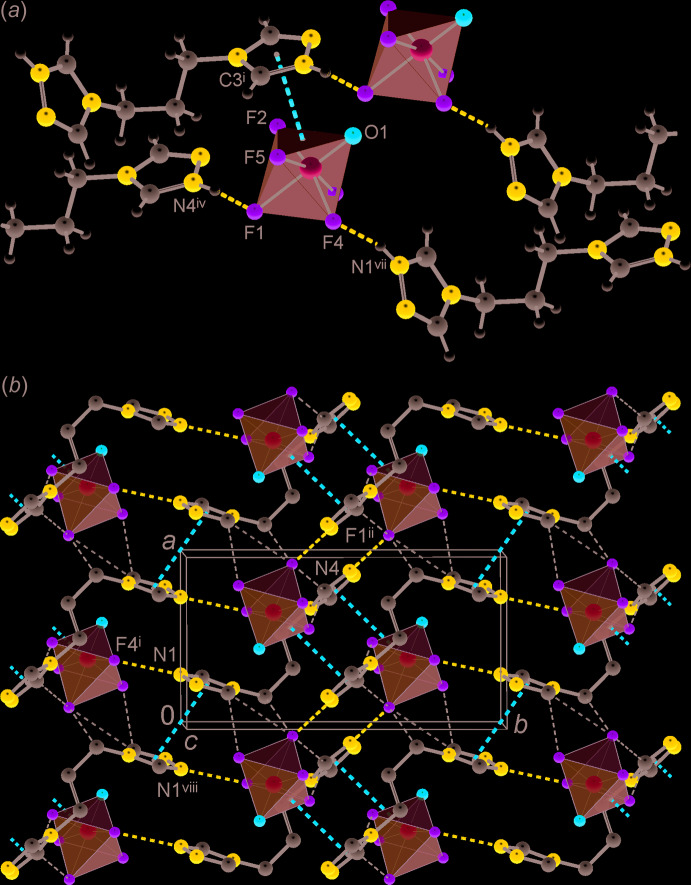
(*a*) Fragment of the double hydrogen-bonded chain showing the *cis*-directing function of the [VOF_5_]^2−^ anions (with respect to the strongest N—H⋯F hydrogen-bond donors) and short anion⋯π contact subtended by the triazole ring to the O1/F2/F5 face of the anion. (*b*) Structure of the hydrogen-bonded layer, viewed nearly down the *c* axis, with the strongest hydrogen bonds and two kinds of stacking inter­actions indicated by blue and red dotted lines, respectively. [Symmetry codes: (i) −*x* + 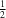, *y* − 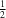, −*z* + 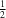; (ii) −*x* + 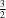, *y* + 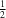, −*z* + 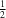; (iv) 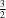 − *x*, −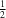 + *y*, 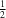 − *z*; (vii) −*x* + 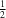, *y* + 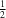, −*z* + 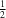; (viii) −*x*, −*y*, −*z*.]

The three-dimensional packing of the title compound is mediated by hydrogen bonding and two kinds of stacking inter­actions. Two strong N—H⋯F hydrogen bonds employ the most underbonded axial F1 atoms of the anion and the *cis*-positioned F4 atom (Fig. 2 ▸). Thus the primary pattern exactly follows the *cis*-directing preferences of the [VOF_5_]^2−^ anions, as suggested by Poeppelmeier and co-workers (Welk *et al.*, 2000 ▸; Gautier *et al.*, 2015 ▸). More distal inter­actions are relevant to weaker CH donors (Table 2 ▸). In total they support nine C—H⋯F contacts with a cut-off-limit of H⋯F = 2.56 Å, which is the sum of the van der Waals radii of these species (Rowland & Taylor, 1996 ▸). The role of the triazole CH groups is notable: in addition to the shortest contacts with the F acceptors [H⋯F = 2.18–2.42 Å], they also form weak C—H⋯N bonds with triazole-N atoms [H⋯N = 2.47 and 2.59 Å; C⋯N = 3.3122 (19) and 3.343 (2) Å]. There are no N/C—H⋯O bonds at all and the shortest H⋯O contact of 2.84 Å considerably exceeds the sum of the corresponding van der Waals radii (2.68 Å; Rowland & Taylor, 1996 ▸). It should be stressed that even such a weak acceptor as the N atom of the cationic moiety is a preferable site for hydrogen bonding, instead of the O atom of the [VOF_5_]^2−^ anion. For the aliphatic portion of the structure, C—H⋯F inter­actions are longer and presumably weaker, whereas shorter H⋯F contacts [2.32 Å] correspond to the triazole-linked methyl­ene groups, as these are more polarized and acidic.

Primary strong N—H⋯F bonding links the ionic counterparts into chains, which aggregate forming layers parallel to the *ab* plane. In a complement to the weak C—H⋯F bonds, these layers are sustained by two types of stacks (Fig. 2 ▸). The first of these may be regarded as an inter­action between the triazolium ring to the F2/F5/O1 face of the anion, with an inter­planar angle of 12.60 (9)° and centroid-to-centroid distance of 3.064 (2) Å. This inter­action is favourable, as a kind of recently recognized anion⋯π bonding (Bauzá *et al.*, 2016 ▸) and it is responsible for the generation of a very short contact: F5⋯C3^i^ = 2.7296 (15) Å [symmetry code: (i) −*x* + 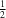, *y* − 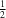, −*z* + 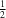]. The second type may concern the stacking of the inversion–related triazolium rings. However, a relatively large inter­centroid distance of 3.626 (2) Å and slippage angle of 64.2 (2)° indicate a lack of overlap (Janiak, 2000 ▸). Taking into account also the zero contribution of C⋯C contacts to the Hirshfeld surface of the cation (see below), one may postulate rather the ion–dipole inter­action of two triazolium N—NH^+^ sites, with the N1⋯N2^viii^ separation of 3.2926 (18) Å [symmetry code: (viii) −*x*, −*y*, −*z*].

**Figure 3:**
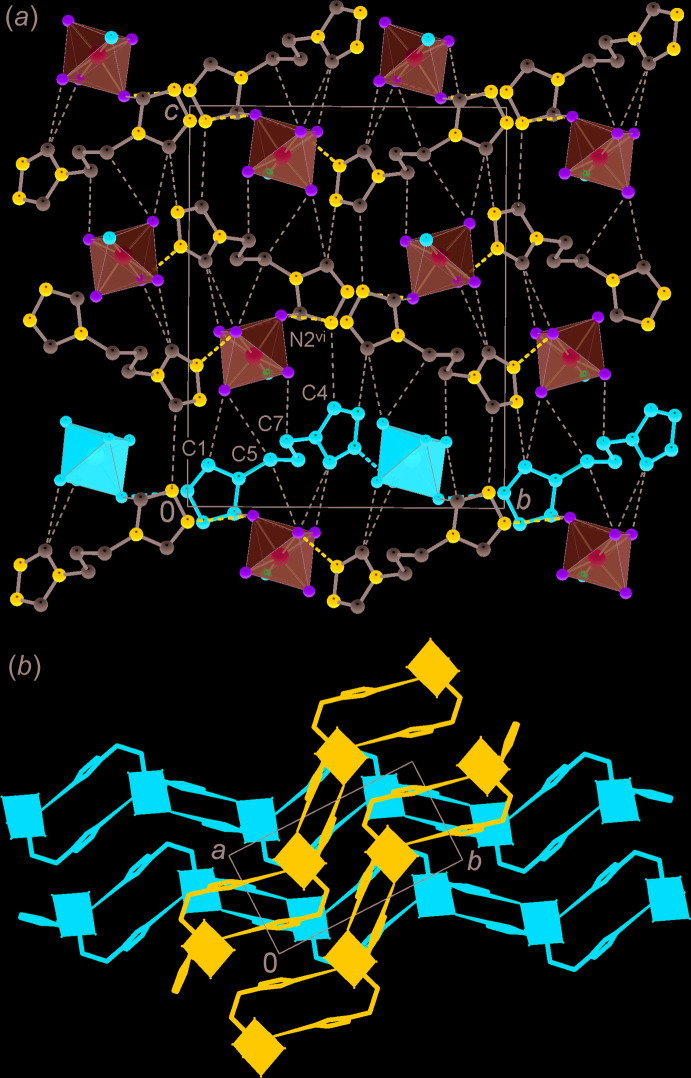
(*a*) Projection of the structure on the *bc* plane showing the extensive C—H⋯F and C—H⋯N inter­actions. A single hydrogen-bonded chain is marked red. (*b*) View down the *c* axis showing the inclined orientation of the hydrogen-bonded chains sustaining adjacent layers. Two separate layers are indicated in blue and red. [Symmetry code: (vi) *x* + 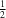, −*y* + 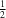, *z* + 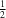.]

The packing of the layers extends the structure in the third dimension. For every next layer of the succession, the direction of the primary N—H⋯F bonded chains is inclined by 56.8° to the direction of chains from the preceding layer (Fig. 3 ▸). Links between the layers represent most of the weak inter­actions, such as C—H⋯N bonds and C—H⋯F bonds with the aliphatic CH donors.

## Hirshfeld analysis   

**Figure 4:**
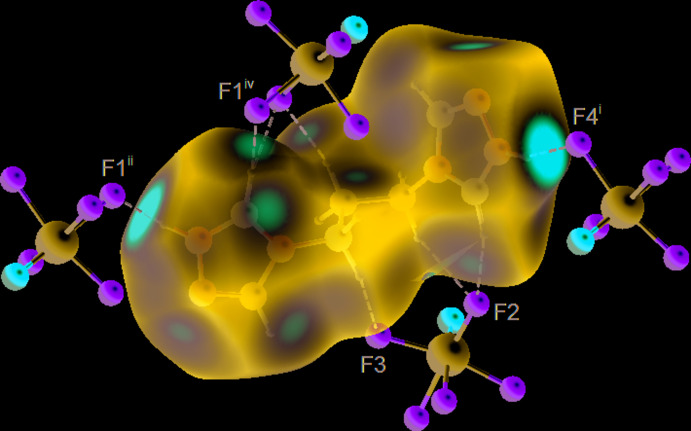
The Hirshfeld surface of the cation mapped over *d*_norm_ in the colour range −0.8385 (red) to 1.3445 (blue) a.u., in the environment of the N—H⋯F and C—H⋯F hydrogen-bonded anions. [Symmetry codes: (i) −*x* + 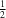, *y* − 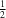, −*z* + 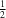; (ii) −*x* + 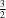, *y* + 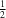, −*z* + 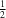; (iv) *x* − 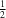, −*y* + 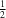, *z* − 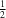.]

**Figure 5:**
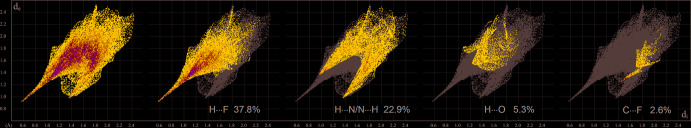
Two-dimensional fingerprint plots for the cations of the title compound, and delineated into the principal contributions of H⋯F, H⋯N/N⋯H, H⋯O and C⋯F contacts. Other important contacts are H⋯H (18.5%), H⋯C/C⋯H (3.4%) and N⋯N (3.0%).

Supra­molecular inter­actions in the title structure were further accessed and visualized by Hirshfeld surface analysis (Spackman & Byrom, 1997 ▸; McKinnon *et al.*, 2004 ▸; Hirshfeld, 1977 ▸; Spackman & McKinnon (2002 ▸) performed with *CrystalExplorer17* (Turner *et al.*, 2017 ▸). The Hirshfeld surface of the cation mapped over *d*_norm_ using a fixed colour scale of −0.8385 (red) to 1.3445 (blue) a.u. indicates a number of red spots related to hydrogen-bond contacts. Particularly prominent spots are associated with the strongest N—H⋯F bonds. However, even the C—H⋯F inter­action with the weakest of the present donors (central CH_2_ group of the tri­methyl­ene linkage) is reflected by a red spot on the surface (Fig. 4 ▸). The contribution of different kinds of inter­atomic contacts to the Hirshfeld surfaces of the individual cations and anions is shown in the fingerprint plots of Figs. 5 ▸ and 6 ▸. Hydrogen-bond inter­actions (H⋯F and H⋯N/N⋯H) account for more than 60% of the contacts of the cations. The strong N—H⋯F bonding is reflected as a very sharp feature pointing to the lower left of the plot, with a shortest contact of 1.5 Å. The more distal H⋯N/N⋯H contacts (22.9%) are identified by a pair of shorter and diffuse spikes. There is no indication of directional H⋯O bonding: the plot represents a rather diffuse collection of points between the above features, with the shortest contact at 2.8 Å. A minor contribution of H⋯O contacts (5.3%) to the entire surface originates in the triazolium/F2,F5,O1 stack, but not in the C—H⋯O inter­actions.

**Figure 6:**
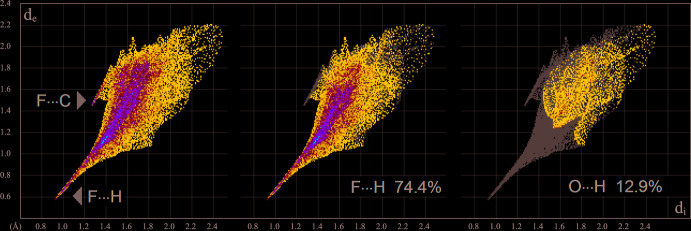
Two-dimensional fingerprint plots for the [VOF_5_]^2−^ anions, showing the very different character of the F⋯H and O⋯H contacts. Very short F⋯C contacts are also readily detectable. Other important contacts are F⋯C(N) and O⋯C(N) contributing 6.8 and 5.4%, respectively.

The same conclusion may be reached when considering the surface area of the [VOF_5_]^2−^ anions. The inter­molecular contacts in this case are overwhelmingly of the type F⋯H (74.4%). In addition to this very sharp spike, the plot clearly reveals the more subtle feature of anion⋯π bonding, which appears as a short spike at 2.7 Å (Fig. 6 ▸). In total, the corres­ponding F⋯C(N) and O⋯C(N) contacts account for 12.2% of the anion contacts. There are no close C⋯C contacts, while the contribution of N⋯N contacts (3.0%) is perceptible in the fingerprint plots of the cations. As noted above, this indicates pairwise ion–dipole inter­actions of the N—NH^+^ fragments, with a lack of genuine π–π inter­actions.

## Synthesis and crystallization   

The bitriazole was prepared in a yield of 33% by the acid-catalysed condensation of 1,3-di­amino­propane and *N*,*N*-di­methyl­formamide azine (Lysenko *et al.*, 2010 ▸). To prepare the title compound, the bitriazole (71.2 mg, 0.40 mmol), V_2_O_5_ (18.2 mg, 0.10 mmol), 0.84 ml of 7% aqueous HF solution (3.0 mmol) and 2 ml of water were placed in a Teflon vessel and heated in a steel bomb at 413 K for 24 h. Cooling to room temperature over a period of 48 h afforded colourless crystals of the title salt, in a yield of 27 mg (40%). Analysis (%) calculated for C_7_H_12_F_5_N_6_OV: C 24.57, H 3.54, N 24.57; found: C 24.38, H 3.49, N 24.70.

## Refinement   

Crystal data, data collection and structure refinement details are summarized in Table 3 ▸. All hydrogen atoms were located and then refined as riding with N—H = 0.87 Å, C—H (triazole) = 0.94 Å and C—H (CH_2_) = 0.98 Å; *U*_iso_(H) = 1.2*U*_eq_(CH) and 1.5*U*_eq_(NH).

## Supplementary Material

Crystal structure: contains datablock(s) global, I. DOI: 10.1107/S205698902000585X/hb7908sup1.cif

Structure factors: contains datablock(s) I. DOI: 10.1107/S205698902000585X/hb7908Isup2.hkl

CCDC reference: 1999654

Additional supporting information:  crystallographic information; 3D view; checkCIF report

## Figures and Tables

**Table 1:** Selected geometric parameters (Å, °)

V1—O1	1.5767 (12)	V1—F3	1.8228 (10)
V1—F5	1.7977 (9)	V1—F4	1.8913 (9)
V1—F2	1.8062 (9)	V1—F1	2.0981 (9)
			
O1—V1—F5	97.49 (6)	F2—V1—F4	165.81 (4)
O1—V1—F2	97.75 (6)	F3—V1—F4	85.92 (4)
F5—V1—F2	91.86 (5)	O1—V1—F1	179.08 (6)
O1—V1—F3	96.57 (6)	C1—N1—N2	111.79 (11)
F5—V1—F3	164.75 (5)	C2—N2—N1	103.46 (12)
F2—V1—F3	92.06 (5)	C3—N4—N5	111.17 (12)
O1—V1—F4	96.44 (6)	C4—N5—N4	104.11 (12)
F5—V1—F4	86.68 (4)		
			
N3—C5—C6—C7	−63.73 (17)	C5—C6—C7—N6	−171.58 (12)

**Table 2:** Hydrogen-bond geometry (Å, °)

*D*—H⋯*A*	*D*—H	H⋯*A*	*D*⋯*A*	*D*—H⋯*A*
N1—H1*N*⋯F4^i^	0.87	1.76	2.6007 (14)	163
N4—H2*N*⋯F1^ii^	0.87	1.64	2.5072 (15)	173
C1—H1⋯F2	0.94	2.37	3.0962 (18)	133
C1—H1⋯N5^iii^	0.94	2.59	3.3122 (19)	134
C2—H2⋯F1^iv^	0.94	2.24	3.0163 (16)	139
C3—H3⋯F1^v^	0.94	2.42	3.2565 (18)	148
C3—H3⋯F5^v^	0.94	2.18	2.9980 (17)	144
C4—H4⋯N2^vi^	0.94	2.47	3.343 (2)	154
C5—H5*A*⋯F2	0.98	2.32	3.2039 (18)	150
C5—H5*B*⋯F1^iv^	0.98	2.54	3.2422 (18)	128
C6—H6*A*⋯F5^v^	0.98	2.50	3.4021 (19)	153
C7—H7*A*⋯F3	0.98	2.47	3.2728 (19)	139
C7—H7*B*⋯F4^v^	0.98	2.54	3.3522 (19)	141

Symmetry codes: (i) 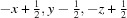; (ii) 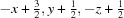; (iii) 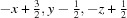; (iv) 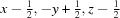; (v) 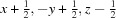; (vi) 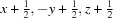.

**Table 3:** Experimental details

Crystal data	
Chemical formula	(C_7_H_12_N_6_)[VOF_5_]
*M*_r_	342.17
Crystal system, space group	Monoclinic, *P*2_1_/*n*
Temperature (K)	213
*a*, *b*, *c* (Å)	6.5915 (4), 12.1969 (10), 15.5669 (10)
β (°)	97.617 (8)
*V* (Å^3^)	1240.47 (15)
*Z*	4
Radiation type	Mo *K*α
μ (mm^−1^)	0.87
Crystal size (mm)	0.25 × 0.22 × 0.20

Data collection	
Diffractometer	Stoe IPDS
Absorption correction	Numerical [*X-RED* (Stoe & Cie, 2001 ▸) and *X-SHAPE* (Stoe & Cie, 1999 ▸)]
*T*_min_, *T*_max_	0.272, 0.303
No. of measured, independent and observed [*I* > 2σ(*I*)] reflections	10733, 2965, 2513
*R*_int_	0.028
(sin θ/λ)_max_ (Å^−1^)	0.663

Refinement	
*R*[*F* ^2^ > 2σ(*F* ^2^)], *wR*(*F* ^2^), *S*	0.028, 0.080, 1.01
No. of reflections	2965
No. of parameters	181
H-atom treatment	H-atom parameters constrained
Δρ_max_, Δρ_min_ (e Å^−3^)	0.37, −0.28

Computer programs: *IPDS Software* (Stoe & Cie, 2000 ▸), *SHELXS97* (Sheldrick, 2008 ▸), *SHELXL2018/1* (Sheldrick, 2015 ▸), *DIAMOND* (Brandenburg, 1999 ▸) and *WinGX* (Farrugia, 2012 ▸).

## supplementary crystallographic information

### Crystal data

**Table:**

(C_7_H_12_N_6_)[VF_5_O]	*F*(000) = 688
*M**_r_* = 342.17	*D*_x_ = 1.832 Mg m^−^^3^
Monoclinic, *P*2_1_/*n*	Mo *K*α radiation, λ = 0.71073 Å
*a* = 6.5915 (4) Å	Cell parameters from 8000 reflections
*b* = 12.1969 (10) Å	θ = 3.2–28.1°
*c* = 15.5669 (10) Å	µ = 0.87 mm^−^^1^
β = 97.617 (8)°	*T* = 213 K
*V* = 1240.47 (15) Å^3^	Prism, colorless
*Z* = 4	0.25 × 0.22 × 0.20 mm

### Data collection

**Table:**

Stoe Image plate diffraction system diffractometer	2513 reflections with *I* > 2σ(*I*)
Radiation source: fine-focus sealed tube	*R*_int_ = 0.028
φ oscillation scans	θ_max_ = 28.1°, θ_min_ = 3.2°
Absorption correction: numerical [X-RED (Stoe & Cie, 2001) and X-SHAPE (Stoe & Cie, 1999)]	*h* = −7→8
*T*_min_ = 0.272, *T*_max_ = 0.303	*k* = −16→16
10733 measured reflections	*l* = −19→19
2965 independent reflections	

### Refinement

**Table:**

Refinement on *F*^2^	Primary atom site location: structure-invariant direct methods
Least-squares matrix: full	Secondary atom site location: difference Fourier map
*R*[*F*^2^ > 2σ(*F*^2^)] = 0.028	Hydrogen site location: difference Fourier map
*wR*(*F*^2^) = 0.080	H-atom parameters constrained
*S* = 1.01	*w* = 1/[σ^2^(*F*_o_^2^) + (0.0603*P*)^2^] where *P* = (*F*_o_^2^ + 2*F*_c_^2^)/3
2965 reflections	(Δ/σ)_max_ < 0.001
181 parameters	Δρ_max_ = 0.37 e Å^−^^3^
0 restraints	Δρ_min_ = −0.28 e Å^−^^3^

### Special details

**Table:**

Geometry. All esds (except the esd in the dihedral angle between two l.s. planes) are estimated using the full covariance matrix. The cell esds are taken into account individually in the estimation of esds in distances, angles and torsion angles; correlations between esds in cell parameters are only used when they are defined by crystal symmetry. An approximate (isotropic) treatment of cell esds is used for estimating esds involving l.s. planes.

### Fractional atomic coordinates and isotropic or equivalent isotropic displacement parameters (Å^2^)

**Table:**

	*x*	*y*	*z*	*U*_iso_*/*U*_eq_	
V1	0.13004 (4)	0.20736 (2)	0.37557 (2)	0.02234 (9)	
O1	−0.0838 (2)	0.25118 (10)	0.33158 (9)	0.0429 (3)	
F1	0.41341 (13)	0.14981 (7)	0.43607 (6)	0.0320 (2)	
F2	0.18077 (15)	0.11480 (7)	0.29038 (6)	0.0361 (2)	
F3	0.28439 (17)	0.31538 (8)	0.33574 (7)	0.0422 (2)	
F4	0.14112 (15)	0.29233 (6)	0.47771 (6)	0.0310 (2)	
F5	0.03593 (14)	0.09762 (7)	0.43649 (6)	0.0329 (2)	
N1	0.27600 (18)	−0.00076 (9)	0.03807 (8)	0.0250 (2)	
H1N	0.317783	−0.068386	0.043251	0.038*	
N2	0.2150 (2)	0.04943 (10)	−0.03920 (8)	0.0290 (3)	
N3	0.19669 (16)	0.16124 (9)	0.07027 (8)	0.0214 (2)	
N4	0.88170 (17)	0.52627 (10)	0.14823 (8)	0.0260 (3)	
H2N	0.961582	0.566665	0.121121	0.039*	
N5	0.8618 (2)	0.53656 (11)	0.23383 (9)	0.0333 (3)	
N6	0.66746 (17)	0.40329 (9)	0.17358 (8)	0.0237 (2)	
C1	0.2642 (2)	0.06477 (11)	0.10324 (10)	0.0262 (3)	
H1	0.296938	0.047489	0.162296	0.031*	
C2	0.1676 (2)	0.14804 (11)	−0.01688 (10)	0.0283 (3)	
H2	0.119140	0.203342	−0.056412	0.034*	
C3	0.7655 (2)	0.44809 (10)	0.11223 (9)	0.0238 (3)	
H3	0.752834	0.426919	0.053704	0.029*	
C4	0.7297 (2)	0.46124 (12)	0.24712 (10)	0.0312 (3)	
H4	0.682993	0.448455	0.300737	0.037*	
C5	0.1521 (2)	0.26078 (11)	0.11809 (10)	0.0261 (3)	
H5A	0.139985	0.240949	0.178200	0.031*	
H5B	0.020264	0.290896	0.092153	0.031*	
C6	0.3158 (2)	0.34849 (11)	0.11766 (10)	0.0270 (3)	
H6A	0.330379	0.367237	0.057561	0.032*	
H6B	0.272302	0.414699	0.145844	0.032*	
C7	0.5216 (2)	0.31175 (11)	0.16388 (10)	0.0274 (3)	
H7A	0.504179	0.282923	0.221218	0.033*	
H7B	0.575771	0.252713	0.130794	0.033*	

### Atomic displacement parameters (Å^2^)

**Table:**

	*U*^11^	*U*^22^	*U*^33^	*U*^12^	*U*^13^	*U*^23^
V1	0.03279 (14)	0.01556 (13)	0.01820 (14)	0.00023 (8)	0.00168 (9)	0.00033 (7)
O1	0.0483 (7)	0.0392 (6)	0.0376 (7)	0.0135 (5)	−0.0074 (5)	0.0005 (5)
F1	0.0311 (4)	0.0312 (4)	0.0318 (5)	0.0036 (3)	−0.0028 (4)	−0.0077 (3)
F2	0.0530 (5)	0.0307 (4)	0.0234 (5)	0.0052 (4)	0.0009 (4)	−0.0081 (3)
F3	0.0674 (7)	0.0296 (4)	0.0319 (6)	−0.0133 (4)	0.0154 (5)	0.0046 (4)
F4	0.0505 (5)	0.0198 (4)	0.0241 (5)	−0.0032 (3)	0.0097 (4)	−0.0041 (3)
F5	0.0405 (5)	0.0260 (4)	0.0308 (5)	−0.0120 (3)	−0.0003 (4)	0.0041 (3)
N1	0.0291 (6)	0.0180 (5)	0.0274 (7)	0.0027 (4)	0.0017 (4)	0.0023 (4)
N2	0.0369 (6)	0.0267 (6)	0.0231 (7)	0.0030 (5)	0.0026 (5)	0.0002 (5)
N3	0.0229 (5)	0.0179 (5)	0.0229 (6)	0.0006 (4)	0.0015 (4)	0.0021 (4)
N4	0.0254 (5)	0.0254 (5)	0.0260 (7)	0.0005 (4)	−0.0005 (4)	0.0025 (5)
N5	0.0408 (7)	0.0314 (6)	0.0256 (7)	−0.0024 (5)	−0.0034 (5)	−0.0038 (5)
N6	0.0277 (5)	0.0211 (5)	0.0217 (6)	0.0023 (4)	0.0009 (4)	0.0009 (4)
C1	0.0306 (7)	0.0219 (6)	0.0248 (8)	0.0025 (5)	−0.0012 (5)	0.0043 (5)
C2	0.0376 (7)	0.0240 (6)	0.0226 (8)	0.0046 (5)	0.0018 (6)	0.0046 (5)
C3	0.0260 (6)	0.0222 (6)	0.0223 (7)	0.0036 (5)	0.0005 (5)	0.0009 (5)
C4	0.0409 (8)	0.0315 (7)	0.0205 (8)	0.0012 (6)	0.0020 (6)	−0.0014 (6)
C5	0.0300 (7)	0.0210 (6)	0.0279 (8)	0.0020 (5)	0.0057 (5)	−0.0026 (5)
C6	0.0329 (7)	0.0177 (6)	0.0291 (8)	0.0017 (5)	−0.0008 (5)	0.0010 (5)
C7	0.0303 (7)	0.0195 (6)	0.0319 (8)	−0.0005 (5)	0.0016 (6)	0.0035 (5)

### Geometric parameters (Å, º)

**Table:**

V1—O1	1.5767 (12)	N5—C4	1.301 (2)
V1—F5	1.7977 (9)	N6—C3	1.3387 (19)
V1—F2	1.8062 (9)	N6—C4	1.3615 (19)
V1—F3	1.8228 (10)	N6—C7	1.4683 (17)
V1—F4	1.8913 (9)	C1—H1	0.9400
V1—F1	2.0981 (9)	C2—H2	0.9400
N1—C1	1.3019 (19)	C3—H3	0.9400
N1—N2	1.3621 (18)	C4—H4	0.9400
N1—H1N	0.8700	C5—C6	1.5201 (19)
N2—C2	1.3014 (19)	C5—H5A	0.9800
N3—C1	1.3361 (16)	C5—H5B	0.9800
N3—C2	1.354 (2)	C6—C7	1.5160 (19)
N3—C5	1.4740 (17)	C6—H6A	0.9800
N4—C3	1.3022 (17)	C6—H6B	0.9800
N4—N5	1.362 (2)	C7—H7A	0.9800
N4—H2N	0.8700	C7—H7B	0.9800
			
O1—V1—F5	97.49 (6)	N1—C1—H1	126.5
O1—V1—F2	97.75 (6)	N3—C1—H1	126.5
F5—V1—F2	91.86 (5)	N2—C2—N3	111.73 (13)
O1—V1—F3	96.57 (6)	N2—C2—H2	124.1
F5—V1—F3	164.75 (5)	N3—C2—H2	124.1
F2—V1—F3	92.06 (5)	N4—C3—N6	107.69 (13)
O1—V1—F4	96.44 (6)	N4—C3—H3	126.2
F5—V1—F4	86.68 (4)	N6—C3—H3	126.2
F2—V1—F4	165.81 (4)	N5—C4—N6	111.47 (14)
F3—V1—F4	85.92 (4)	N5—C4—H4	124.3
O1—V1—F1	179.08 (6)	N6—C4—H4	124.3
F5—V1—F1	82.13 (4)	N3—C5—C6	112.85 (12)
F2—V1—F1	83.11 (4)	N3—C5—H5A	109.0
F3—V1—F1	83.73 (5)	C6—C5—H5A	109.0
F4—V1—F1	82.71 (4)	N3—C5—H5B	109.0
C1—N1—N2	111.79 (11)	C6—C5—H5B	109.0
C1—N1—H1N	124.1	H5A—C5—H5B	107.8
N2—N1—H1N	124.1	C7—C6—C5	112.37 (11)
C2—N2—N1	103.46 (12)	C7—C6—H6A	109.1
C1—N3—C2	106.01 (12)	C5—C6—H6A	109.1
C1—N3—C5	127.58 (13)	C7—C6—H6B	109.1
C2—N3—C5	126.34 (12)	C5—C6—H6B	109.1
C3—N4—N5	111.17 (12)	H6A—C6—H6B	107.9
C3—N4—H2N	124.4	N6—C7—C6	110.88 (11)
N5—N4—H2N	124.4	N6—C7—H7A	109.5
C4—N5—N4	104.11 (12)	C6—C7—H7A	109.5
C3—N6—C4	105.54 (12)	N6—C7—H7B	109.5
C3—N6—C7	127.50 (12)	C6—C7—H7B	109.5
C4—N6—C7	126.96 (13)	H7A—C7—H7B	108.1
N1—C1—N3	107.01 (12)		
			
C1—N1—N2—C2	0.38 (16)	C7—N6—C3—N4	−179.39 (12)
C3—N4—N5—C4	0.12 (16)	N4—N5—C4—N6	0.68 (17)
N2—N1—C1—N3	−0.72 (16)	C3—N6—C4—N5	−1.21 (16)
C2—N3—C1—N1	0.74 (15)	C7—N6—C4—N5	179.41 (12)
C5—N3—C1—N1	177.92 (12)	C1—N3—C5—C6	104.60 (16)
N1—N2—C2—N3	0.11 (16)	C2—N3—C5—C6	−78.76 (17)
C1—N3—C2—N2	−0.54 (17)	N3—C5—C6—C7	−63.73 (17)
C5—N3—C2—N2	−177.76 (13)	C3—N6—C7—C6	−75.14 (17)
N5—N4—C3—N6	−0.88 (15)	C4—N6—C7—C6	104.11 (16)
C4—N6—C3—N4	1.23 (14)	C5—C6—C7—N6	−171.58 (12)

### Hydrogen-bond geometry (Å, º)

**Table:**

*D*—H···*A*	*D*—H	H···*A*	*D*···*A*	*D*—H···*A*
N1—H1*N*···F4^i^	0.87	1.76	2.6007 (14)	163
N4—H2*N*···F1^ii^	0.87	1.64	2.5072 (15)	173
C1—H1···F2	0.94	2.37	3.0962 (18)	133
C1—H1···N5^iii^	0.94	2.59	3.3122 (19)	134
C2—H2···F1^iv^	0.94	2.24	3.0163 (16)	139
C3—H3···F1^v^	0.94	2.42	3.2565 (18)	148
C3—H3···F5^v^	0.94	2.18	2.9980 (17)	144
C4—H4···N2^vi^	0.94	2.47	3.343 (2)	154
C5—H5*A*···F2	0.98	2.32	3.2039 (18)	150
C5—H5*B*···F1^iv^	0.98	2.54	3.2422 (18)	128
C6—H6*A*···F5^v^	0.98	2.50	3.4021 (19)	153
C7—H7*A*···F3	0.98	2.47	3.2728 (19)	139
C7—H7*B*···F4^v^	0.98	2.54	3.3522 (19)	141

Symmetry codes: (i) −*x*+1/2, *y*−1/2, −*z*+1/2; (ii) −*x*+3/2, *y*+1/2, −*z*+1/2; (iii) −*x*+3/2, *y*−1/2, −*z*+1/2; (iv) *x*−1/2, −*y*+1/2, *z*−1/2; (v) *x*+1/2, −*y*+1/2, *z*−1/2; (vi) *x*+1/2, −*y*+1/2, *z*+1/2.
